# Dual Therapy in Inflammatory Bowel Disease

**DOI:** 10.3390/biom15020222

**Published:** 2025-02-03

**Authors:** Gabriele Altieri, Alessandra Zilli, Tommaso Lorenzo Parigi, Mariangela Allocca, Federica Furfaro, Gionata Fiorino, Clelia Cicerone, Laurent Peyrin-Biroulet, Silvio Danese, Ferdinando D’Amico

**Affiliations:** 1 Gastroenterology and Endoscopy, IRCCS San Raffaele Hospital, 20132 Milan, Italy; altieri.gabriele@hsr.it (G.A.);; 2 Faculty of Medicine and Surgery, Vita Salute San Raffaele University, 20132 Milan, Italy; 3 Gastroenterology and Digestive Endoscopy, San Camillo-Forlanini Hospital, 00152 Rome, Italy; 4 Department of Gastroenterology, INFINY Institute, INSERM NGERE, CHRU de Nancy, Université de Lorraine, F-54500 Vandœuvre-lès-Nancy, France

**Keywords:** inflammatory bowel disease, Crohn’s disease, ulcerative colitis, dual-targeted therapy, combination therapy

## Abstract

Inflammatory bowel diseases (IBDs), including ulcerative colitis (UC) and Crohn’s disease (CD), are chronic and complex autoimmune conditions. Despite the advancements in biologics and small molecules, the therapeutic ceiling persists, posing significant treatment challenges and contributing to the concept of difficult-to-treat IBD. Dual-targeted therapy (DTT), combining two biologic agents or biologics with small molecules, has emerged as a novel approach to address this unmet need by targeting multiple inflammatory pathways simultaneously. Evidence suggests that DTT holds promise in improving clinical and endoscopic outcomes, especially in patients with refractory disease or extraintestinal manifestations. Safety data, while consistent with monotherapy profiles, highlight the importance of vigilant monitoring for infections and other adverse events. Continued research and high-quality trials are crucial to defining optimal DTT regimens and broadening its clinical applicability. This review explores the efficacy and safety of DTT in IBD, reporting data from clinical trials, systematic reviews, and real-world studies.

## 1. Introduction

Inflammatory bowel diseases (IBDs) are chronic, autoimmune, and life-impairing conditions that affect the gastrointestinal tract, comprising two different entities: ulcerative colitis (UC) and Crohn’s disease (CD) [1,2,3]. Over the years, the therapeutic landscape in IBD management has undergone a significant evolution, progressing from conventional treatments like mesalamine, corticosteroids, and immunosuppressants to the advent of biologic agents (including anti-tumor necrosis factor α, anti-α4β7 integrin biologic agents, anti-interleukin (IL)-12-23, and selective IL23 inhibitors) and small-molecule drugs (SMDs) (Janus kinase (JAK) inhibitors and Sphingosine 1-phosphate (S1P) modulators), offering more targeted and effective options [4,5]. Nevertheless, IBD still presents significant treatment challenges, with patients often facing issues with inadequate response and unsatisfactory disease remission. Despite all these pharmacological breakthroughs and improvements, remission rates do not surpass an upper limit of 30–50% in induction trials [6,7]. Furthermore, patients who do not respond to first-line biologic therapy often face diminished outcomes with subsequent therapies, contributing to the complex concept of “difficult-to-treat IBD” [8,9]. In this challenging context, dual-targeted therapy (DTT), which combines two or more targeted therapies (such as biologics and small-molecule drugs), emerges as a promising strategy. This approach aims to target multiple inflammatory pathways simultaneously, inspired by successful combination strategies in other medical fields, such as oncology [10], cardiology [11], neurology [12], and infectiology [13,14]. DTT offers the potential to overcome the limitations of monotherapy and enhancing clinical outcomes, hoping to break the therapeutic ceiling [15]. This narrative review aims to provide a thorough overview of the efficacy and the safety of DTT in the treatment of IBD.

## 2. Efficacy of DTT

DTT, although not yet included in current guidelines, is one of the most extensively researched areas in the management of IBD. The rationale behind DTT lies in the possibility to target multiple inflammatory pathways simultaneously [16,17,18,19], thus offering a broader therapeutic impact and potentially synergistic effects that could lead to better outcomes [20,21]. Often, DTT is looked upon as a promising strategy in all the patients showing difficult-to-treat disease stigmata, like refractoriness to multiple biologic therapies, concomitant extra intestinal manifestations (EIMs) or other autoimmune diseases, necessity for surgery, and many more [22].

A significant application of DTT in IBD is its use in managing EIMs [23,24]. DTT offers a unique advantage in addressing the broader systemic nature of IBD: this tailored approach acknowledges the complex, multi-systemic nature of IBD and expands the therapeutic possibilities for patients whose disease burden extends beyond the gastrointestinal tract [25,26,27]. Data from real-world practice increasingly support the use of DTT in IBD, even though the number of randomized controlled trials (RCTs) still lags.

### 2.1. Efficacy of DTT in UC

Chronologically, the earliest instance of combination therapy in UC involved the combination of an immunosuppressant drug with a biologic agent targeting tumor necrosis factor alpha (TNF). The efficacy of such a combination has been tested in an RCT by Panaccione et al. (2014) [28]: after 16 weeks of observation, 39.7% (31 of 78) of patients achieved corticosteroid-free remission after receiving a combination of infliximab and azathioprine compared to 22.1% (17 of 77; *p* = 0.017) receiving only infliximab and 23.7% (18 of 76; *p* = 0.032) receiving azathioprine alone. Expanding on this topic, another RCT by Roblin et al. (2020) [29] proved that in patients who already failed to achieve remission using a first-line anti-TNF drug, the combination of an anti-TNF agent and azathioprine led to higher rates of remission than the biologic alone. This effect is probably linked to the previously mentioned targeting of multiple inflammatory pathways and to the reduction of anti-biologic antibody formation [30]. Thus, this strategy is recommended by the most recent European Crohn’s and Colitis Organization (ECCO) guidelines [5].

More recently, research in UC management is progressing from conventional therapy, which typically involves an immunosuppressant combined with a biologic, to DTT for its potential applications in difficult-to-treat settings. To date, the only RCT regarding DTT in UC is the VEGA study (2023) [31]: a randomized, double-blind, and controlled trial conducted across 54 sites in nine countries. This trial was designed to evaluate the efficacy of guselkumab plus golimumab in UC compared to either monotherapy. Adults (from 18 up to 65 years) who had a confirmed diagnosis of moderately-to-severely active UC (Mayo score 6–12) with a baseline endoscopy subscore of 2 or higher were eligible for inclusion. A total of 214 patients were proportionally allocated to three groups via randomization: combination therapy, guselkumab monotherapy, or golimumab monotherapy. As for the primary endpoint, a larger number of patients in the DTT group achieved clinical response (golimumab + guselkumab, 83%; golimumab, 61%, *p* = 0.0032; guselkumab, 75%, *p* = 0.2155) at 12 weeks compared to both the monotherapy groups. At the same timepoint, clinical remission rates followed a similar trend, with the combination group showing higher percentages (golimumab + guselkumab, 37%; golimumab, 22%, *p* = 0.0578; guselkumab, 21%, *p* = 0.0412). Similarly, by week 38, the DTT group continued to outperform the monotherapy groups in both clinical response and remission rates. Endoscopic findings further supported these outcomes, with a greater proportion of patients in the DTT group showing endoscopic and histological improvements at week 38.

A broader perspective on the current state of the art in DTT is provided by a systematic review and meta-analysis by Ahmed et al. (2022) [32], focusing on the utilization of DTT in IBD patients. This analysis included data from 30 studies, predominantly observational, encompassing both UC and CD patients. The total pooled population consisted of 279 patients, 22% of whom had UC. The primary indications for starting DTT was medically refractory disease (81%), with a median number of two biologic drugs used prior to DTT. The most frequently utilized combination therapies were a TNF antagonist paired with an anti-integrin agent, followed by an anti-integrin agent combined with ustekinumab. Additionally, 20% of the studies included combinations of biologic agents and SMDs. At a median follow-up of 32 weeks, the pooled clinical response and remission rates were 72% and 52%, respectively, while the endoscopic response and remission rates were 58% and 33%, respectively. Specifically, among UC patients, 56% achieved clinical response, and 44% reached clinical remission. Despite these promising results, the heterogeneity of study designs and outcome measures across the included studies further solidifies the need for high-quality RCTs to validate the potential of DTT in clinical practice.

Although significant progress has been made in exploring DTT in UC, the optimal combination of biologics or SMDs remains uncertain. However, the choice of which combination to employ is often guided by individualized patient factors, such as prior treatment failures or disease characteristics. Therefore, in a recent systematic review, Berinstein et al. (2023) [33] focused on the various pharmacological combinations seen throughout the years of DTT experimentation. The most prevalent combination was anti-TNF and anti-integrin, documented in 113 of the 288 total patients. Despite the vast majority of data coming from CD cases, the efficacy of this combination showed its potential in the field of UC. For instance, in a prospective cohort described by Buer et al. (2018) [34], six UC patients were treated with infliximab plus vedolizumab for a median of 6 months. At the end of the follow-up (12–18 months), clinical remission was reported in all six of the patients. Moreover, endoscopic remission was achieved in three of them, while the other three patients showed significant improvement nonetheless. Additionally, Berinstein et al.’s work outlined other combinations of drugs featuring tofacitinib [35,36,37]. Regarding this topic, Scheinberg et al. (2020) [38] reported data from eight UC patients treated with a combination of vedolizumab and tofacitinib for a median duration of 6 months: 62.5% had a clinical response defined as patient-reported improvement in symptoms. These results were substantially different from the aforementioned prospective study by Buer and colleagues [34]; the lack of homogeneity among studies described by Berinstein and colleagues does not permit an objective evaluation of efficacy differences between drug combinations. Further investigation is needed to better understand the efficacy and feasibility of the various drug combinations.

As previously mentioned, DTT is often employed in cases of complex EIMs in UC patients. In Ahmed and colleagues’ systematic review and meta-analysis [32], 12% of the included patients received DTT due to concomitant EIMs. Two recent case reports showed the efficacy of combining vedolizumab with TNF inhibitors (infliximab and golimumab) in treating UC combined with pyoderma gangrenosum [39] and spondylarthritis [40], respectively. Additionally, another case report by Privitera et al. (2021) [24] followed three UC patients who started DTT due to uncontrolled EIMs. In these patients, vedolizumab was combined with certolizumab in a case of concomitant spondylarthritis and with ustekinumab and secukinumab, respectively, in two cases of psoriatic disease. All three patients had promising results in both intestinal manifestations and EIMs at 2- and 6-month follow-ups.

### 2.2. Efficacy of DTT in CD

Similarly to UC, the only combination therapy recommended in recent CD guidelines involves an anti-TNF drug, like infliximab or adalimumab, and a thiopurine. The efficacy of such a combination was evaluated in the SONIC trial [41], where 96 of 169 (56.8%) CD patients achieved clinical remission by week 26 compared to 75 patients (44.4%) receiving infliximab alone (*p* = 0.02) and 51 patients (30.0%) receiving azathioprine alone (*p* < 0.001). Therefore, it is recommended in the most recent ECCO guidelines [4].

As research started shifting toward DTT, the first randomized, placebo-controlled trial regarding this strategy was conducted by Sands et al. (2007) [42]. The study aimed to assess whether combining infliximab and natalizumab (a humanized monoclonal antibody targeting α4-integrin) could improve outcomes in patients with active CD who had not achieved remission with ongoing infliximab therapy alone. A total of 79 patients were enrolled and randomized in a 2:1 ratio to receive either natalizumab (300 mg every 4 weeks; n = 52) or a placebo (n = 27) alongside infliximab (5 mg/kg every 8 weeks). The participants had a mean disease duration of approximately 10 years and presented with high baseline Crohn’s Disease Activity Index (CDAI) scores (243.6 in the placebo group and 263.8 in the natalizumab group).

While the study results did not reach statistical significance, they revealed notable trends suggesting potential benefits of the combination therapy. The mean CDAI score decreased by 37.7 points in the natalizumab group compared to a reduction of 3.5 points in the placebo group (p = 0.084). Furthermore, clinical remission (CDAI < 150) was achieved in 46% of patients receiving natalizumab compared to 41% in the placebo group. Improvements in quality of life, as measured using the Inflammatory Bowel Disease Questionnaire (IBDQ), and patient-reported outcomes were modestly more pronounced in the natalizumab group. Nowadays, however, natalizumab is rarely used due to the high risk of progressive multifocal leukoencephalopathy [43].

Although the small sample size limited the statistical power of the study, these findings marked an important milestone: they suggested that overlapping biologic therapies could be a feasible approach, paving the way for the eventual development of DTT as a therapeutic strategy in IBD.

Yang et al. (2020) [44] investigated DTT in refractory CD, offering critical insights into its potential efficacy and safety. Among the 22 patients with refractory CD undergoing DTT trials, the majority had stricturing or penetrating disease phenotypes and extensive prior treatment failures (median of four biologics). The most common combinations included vedolizumab paired with ustekinumab or TNF antagonists due to their known safety and effectiveness [4,45].

In terms of efficacy, 43% of patients achieved endoscopic improvement, and 26% reached endoscopic remission. Clinical response and remission were observed in 50% and 41% of cases, respectively, with significant reductions in the Simple Endoscopic Score for CD (SES-CD) (from 14.0 to 6.0, *p* = 0.0005), Crohn’s disease patient-reported outcome-2 score (PRO-2), and C-reactive protein (CRP) levels. Perianal fistula activity also improved, decreasing from 50% at baseline to 33% post-treatment, a noteworthy result due to the recognized complexity and aggressiveness of the penetrating phenotype [46]. A recent retrospective analysis [47] described similar findings regarding endoscopic response and remission (51.7% and 27.6%, respectively) in 42 CD patients undergoing DTT; however, the clinical remission rate was much higher than in the previously mentioned Yang et al. study [44], reaching 57.4% at a median follow-up of 13 months, defined as Harvey–Bradshaw Index score ≤ 4. Similar results were described by Hassan et al. [48] in a retrospective study from 2025, where 16 of 21 (76%) refractory CD patients, treated with a combination of ustekinumab + vedolizumab, achieved clinical remission (described as a Harvey–Bradshaw Index score ≤ 4) at the end of the 52-week follow-up. These results suggest that DTT may offer a feasible option for patients with severe disease who fail single-biologic therapies. The findings underscore the need for larger, controlled studies to confirm these results and especially to determine the ideal approach for selecting dual-biologic regimens.

Moreover, a recent study by Miyatani et al. (2024) [49] evaluated 10 patients with refractory CD, of whom 80% had previous surgical interventions, a median of four prior biologic exposures, and multiple failed therapies. The majority (80%) of patients receiving DTT had ustekinumab as a baseline therapy, with upadacitinib added subsequently to address ongoing disease activity or EIMs. The combination resulted in clinical remission for 83% of patients with active CD and an improvement in symptoms for 75% of those with EIMs, such as joint pain. The rapid and significant responses seen with this combination underscore the potential for DTT to achieve robust disease control in patients with limited options. Even though the results are promising, the authors also acknowledged that further studies are needed to determine optimal dosing regimens, the timing of interventions, and whether de-escalation strategies could maintain remission once achieved.

Lastly, expanding on the concept of conventional combination therapy, as described in the current guidelines [4], there have been examples of trials using a combination of three drugs. The EXPLORER trial [50] (a prospective, phase 4, single-arm, open-label study) aimed at evaluating the efficacy and safety of triple-combination therapy with vedolizumab, adalimumab, and methotrexate in biologic-naïve patients with newly diagnosed, moderate- to high-risk CD. The study included 55 patients with a diagnosis of CD within 24 months prior to screening, moderate to severe disease activity (defined by a SES-CD] ≥ 7 for colonic disease or ≥4 for isolated ileal disease), and no prior exposure to biologic therapies. The participants received an induction regimen of vedolizumab, adalimumab, and methotrexate. The primary endpoint was endoscopic remission at week 26, defined as SES-CD ≤ 2. Secondary endpoints included clinical remission (CDAI < 150), endoscopic response (≥50% reduction in SES-CD from baseline), and changes in inflammatory biomarkers such as CRP and fecal calprotectin. At week 26, 34.5% (95% confidence interval [CI], 21.1–48.0) of the enrolled patients achieved endoscopic remission, with a higher rate of 42.2% (95% CI, 26.7–57.8) among the observed cases who completed a colonoscopy. Clinical remission was observed in 61.8% (95% CI, 48.1–75.6) at week 10 and 54.5% (95% CI, 40.5–68.6) at week 26, demonstrating robust early and sustained symptom control. Additionally, 56.4% (95% CI, 42.3–70.4) achieved endoscopic response, and the mean SES-CD reduction from baseline was 8.9 points. The authors highlighted the significance of these results in the context of prior benchmarks for biologic monotherapies. A post hoc Bayesian analysis estimated a 99.99% probability that the triple-combination therapy provided superior efficacy to placebo. Moreover, the probabilities of exceeding remission rates for vedolizumab (27%) and adalimumab (30%) monotherapies were 86.3% and 71.4%, respectively. The findings underline the potential synergistic effect of combining biologics with distinct mechanisms of action, although there is a persistent need for larger controlled trials to confirm these observations.

A summary of the selected evidence regarding DTT efficacy is displayed in Table 1.

## 3. Safety of DTT

The safety of DTT in IBD is a particularly critical consideration. Given that all biologic treatments inherently modulate immune system activities [51], this effect is potentially magnified when combining biologic agents [52]. Even though there have been some reports from the rheumatologic literature regarding some uncertainties with DTT [53], the availability of safe and selective advanced therapies [54] could be pivotal for the utilization of DTT in IBD.

### 3.1. Safety of DTT in UC

In the previously mentioned VEGA study [31], the safety of DTT was monitored throughout the 50 weeks follow-up period, during which potential adverse events (AEs) were collected. The authors found a comparable safety profile between the DTT and monotherapies. By week 50, AEs were reported in 63% of DTT recipients, 76% with golimumab monotherapy, and 65% with guselkumab monotherapy. Infections occurred in 31%, 32%, and 24% of patients respectively, while serious infections were similarly rare across groups (≈3%). Notably, serious AEs leading to treatment discontinuation were slightly higher with combination therapy (10%) versus golimumab (6%) and guselkumab (1%). Importantly, regarding AEs of special interest, malignancies were reported only in the guselkumab group (regarding only 1 of 71 patients, with no demonstrable association with the administered drug), and one tuberculosis case was observed with combination therapy. Two deaths occurred during the study: one in the combination therapy group due to poisoning of unknown origin and one in the guselkumab monotherapy group caused by COVID-19. Overall, the dual therapy demonstrated manageable safety, aligning with individual treatments.

The existing literature on DTT primarily explores the safety of the specific biologic combinations, offering insights that apply across general cohorts of IBD patients. A recent systematic review by Alayo et al. (2022) [55] reported several safety outcomes of DTT in UC. Among the 75 UC patients analyzed, the most commonly studied combinations included vedolizumab with an anti-TNF drug and tofacitinib with vedolizumab. AEs occurred in approximately 18–24% of patients across various combinations, with serious adverse events (SAEs) observed in about 1–10%. Across all patients included in the various studies, infections emerged as the primary adverse events recorded during the follow-up period, reaffirming prior concerns regarding the heightened risk of infection associated with combination therapy [23]. The primary SAEs involved infections such as *Clostridioides difficile* and bacterial pneumonia. These findings underlined a relatively low risk of SAEs in UC patients undergoing DTT, aligning with the safety profiles of monotherapy trials for these agents.

Despite the limited availability of data from meta-analyses and systematic reviews, there is an abundance of data derived from real-world studies, including small case series. In the previously cited Buer et al. prospective study [34], during the follow-up period, which lasted a median of 17 months (range 12–20 months), the combination therapy demonstrated an acceptable safety profile. Notably, no infusion reactions occurred, and no patient experienced severe or unexpected adverse events. The reported adverse events in UC patients included two cases of upper respiratory tract infections (tonsillitis and sinusitis), both treated with antibiotics and resolved without further complications. After the discontinuation of anti-TNF therapy, additional adverse events were observed in individual patients, including dyspnea, monoarthritis, and tendinitis. Dyspnea, occurring five months into combination treatment, resolved spontaneously without requiring intervention. Monoarthritis and tendinitis were managed successfully with short-term corticosteroid therapy. No UC patients experienced serious infections, malignancies, or other significant complications during the study period.

Another prospective cohort study, conducted by Gilmore et al. (2021) [56], demonstrated that the combination therapy of infliximab and tofacitinib was well tolerated in a small cohort of five patients with severe refractory UC despite their aggressive disease phenotype. Safety findings over a median follow-up of nine months revealed minimal adverse events. Notably, one patient developed varicella zoster, successfully managed with valaciclovir and a temporary interruption of tofacitinib. Importantly, no severe complications, such as thromboembolism, hypercholesterolemia, or significant infections requiring hospitalization, were reported.

Furthermore, a retrospective study by Goessens et al. (2021) [57] analyzed 98 patients receiving dual therapy for IBD, including 40 patients with UC, offering additional insights. The cohort predominantly included individuals with longstanding and refractory disease, a median disease duration of 10 years, and extensive prior exposure to biologics and small molecules. Various combinations of therapies were employed, most frequently involving anti-TNF agents, anti-integrins, anti-IL agents, and Janus kinase inhibitors. A total of 42 adverse events were reported across 122 patient-years of follow-up. Among UC patients, serious infections were uncommon, and no deaths or new cancer diagnoses were noted. Notably, infections requiring hospitalization occurred in approximately 10% of the overall cohort, often linked to concomitant corticosteroid or immunomodulator use, highlighting the need for caution in managing such patients. No specific safety differences between UC and CD were identified. Another recent retrospective study [58] analyzed the safety of dual-targeted therapy in 42 UC patients with severe refractory disease. Serious adverse events were uncommon, with no deaths or thromboembolic events recorded during a median follow-up of 39.1 weeks. The most frequent complications were mild infections, primarily upper respiratory tract (eight cases), and dermatologic manifestations. Importantly, therapy-related discontinuations were rare, with only five cases due to adverse effects. Overall, the safety profile was acceptable, underscoring the low risk of severe complications, even in a high-risk cohort.

### 3.2. Safety of DTT in CD

CD characteristics like penetrating disease, the need for multiple surgical procedures, and surgery-associated hospitalization increase the risk for infections in CD patients [59,60,61]. This heightened vulnerability underscores the critical importance of assessing infection risks associated with DTT.

In the previously cited Sands et al.’s RCT [42], the safety outcomes revealed comparable AE rates between the combination therapy group (92%) and the infliximab-only group (100%). Common AEs included headaches, CD exacerbation, nausea, and nasopharyngitis, with infection rates similar across groups (27% vs. 30%). SAEs were infrequent, with only two cases of intestinal obstruction reported, neither deemed related to the therapies. Notably, no opportunistic infections or malignancies occurred during the study. Immunogenicity was low, with 4% of patients developing anti-natalizumab antibodies and 13% developing anti-infliximab antibodies. The authors highlighted the absence of significant safety concerns in this short-term trial, particularly no drug–drug interactions or hypersensitivity reactions to natalizumab.

Additionally, Ahmed and colleagues’ systematic review [32] reinforced the concept that safety is a critical factor in dual therapy, with AEs reported in approximately 31% of cases and serious SAEs in 6.5%. While these rates are consistent with monotherapy data, infections (20%) and malignancy (1.6%) rates across the included studies raised concerns about cumulative immunosuppressive effects. The authors noted that combinations involving vedolizumab and ustekinumab showed promising safety profiles due to their gut-selective or cytokine-specific mechanisms, mitigating systemic immune modulation.

In Miyatani et al. study [49], adverse events were noted in 40% of cases, primarily mild infections (i.e., respiratory or sinus infections) that resolved without hospitalization. However, one patient experienced a bowel obstruction requiring hospitalization, and another discontinued therapy due to nausea and a cutaneous fungal infection. Nine out of ten patients continued therapy during the median 10-month follow-up: this suggests acceptable tolerability in a smaller cohort (even though discontinuation due to adverse effects occurred in one case), bolstering the role of DTT in these conditions. The authors also emphasized the lack of severe infections, consistent with findings for ustekinumab and upadacitinib in the literature [62,63].

Lastly, in the EXPLORER trial [50], safety was one of the primary concerns due to the application of three drugs simultaneously. During the 26 weeks of observation, AEs occurred in 87.3% of patients, with most being mild or moderate in severity. SAEs were observed in 10.9% of patients, including small intestine obstruction and infections such as perirectal abscesses. Notably, no new safety signals emerged; the incidence of AEs and SAEs was comparable to that observed with monotherapy using the individual agents. Discontinuation due to AEs was infrequent, highlighting the regimen’s tolerability.

A summary of selected evidence regarding DTT safety is displayed in Table 2.

## 4. Data from Other Immune-Mediated Inflammatory Disorders

A systematic review and meta-analysis by Solitano et al. (2024) [64] investigated the efficacy and safety of DTT in immune-mediated inflammatory diseases (IMIDs).

The authors systematically evaluated RCTs, comparing DTT with monotherapy in IMIDs, including rheumatoid arthritis (RA) and systemic lupus erythematosus (SLE). The meta-analysis encompassed ten trials, enrolling a total of 1154 patients. Eight trials were conducted in rheumatology and two in gastroenterology, focusing on the induction of clinical remission as the primary outcome, along with safety endpoints such as AEs and SAEs. In rheumatology, DTT did not demonstrate significant benefits over monotherapy for clinical remission in RA (pooled RR, 1.75; 95% CI, 0.60–5.13; moderate heterogeneity, I^2^ = 33%) or SLE (RR, 1.20; 95% CI, 0.53–2.72). Similarly, no improvement was observed with DTT in secondary outcomes, such as the American College of Rheumatology 20 and 50 Response Criteria (ACR20 and ACR50) response rates. Notably, patients with RA in the DTT arms experienced a higher incidence of AEs like infections, dermatological reactions, and musculoskeletal symptoms (RR, 1.07; 95% CI, 1.01–1.12), although there was no significant difference in the SAEs compared to monotherapy. These findings align with observations from the rheumatology literature, suggesting that DTT may exacerbate risks without adding clinical benefits, probably due to overlapping immune mechanisms.

The authors hypothesize that the substantial differences between DTT outcomes in rheumatology and gastroenterology may stem from variations in disease mechanisms and immune targets, as well as differences in the safety profiles of the combinations used. Moreover, RA and SLE patients are more prone to cardiovascular, respiratory, and systemic comorbidities [65,66] compared to usually younger IBD patients, partially explaining the safety differences. In IBD, combination therapies targeting complementary inflammatory pathways may exert synergistic effects, as supported by transcriptomic analyses [67] from studies like VEGA [31].

## 5. Discussion

**Figure 1:**
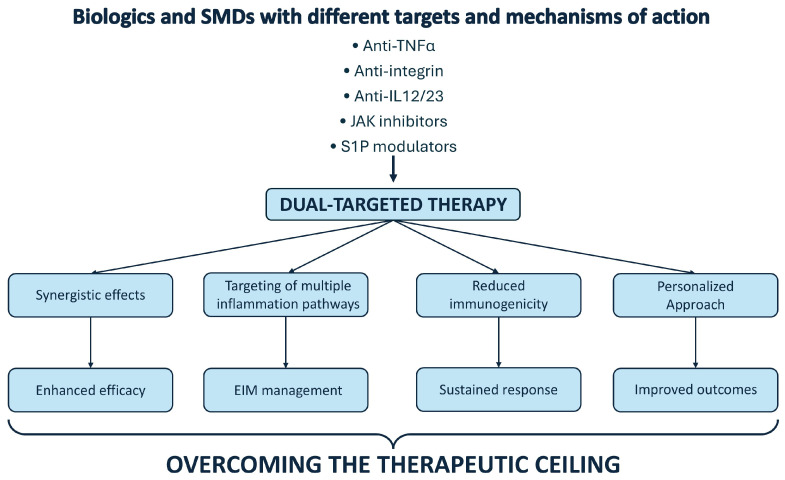
Key benefits of DTT in overcoming the therapeutic ceiling in IBD.

The emergence of dual-targeted therapy DTT represents a paradigm shift in the management of IBD, particularly for patients with refractory disease who fail to achieve remission with standard monotherapies [68]. While significant advances in biologics and small molecules have expanded the therapeutic arsenal, a substantial proportion of patients remain inadequately controlled, highlighting the need for innovative strategies. DTT, by targeting distinct yet complementary immune pathways, offers the potential to overcome the therapeutic ceiling observed with single-agent approaches [69] (Figure 1).

While targeting multiple pathophysiological pathways is a functional strategy, as already proven in many other branches of medicine, choosing the most optimal combination therapy for a specific individual is crucial in managing resources and improving the overall outcomes. An important part of research in IBD is exploring the field of molecular targets and how different inflammatory mediators interact with each other. Currently available data describe the intercorrelation between IL-23 and TNF-α: the latter appears directly correlated to IL-23 production because of the activation of Th17 lymphocytes [70,71,72]. Thus, these findings support the combination of an anti-TNF agent with an anti-IL-23 drug because of the synergistic effect in reducing the total amount of circulating TNF-α, bolstering the results of the VEGA study [31]. Moreover, with the continuous expansion of the therapeutic arsenal and the advent of drugs like risankizumab and mirikizumab (both being p19-directed IL-23 monoclonal antibodies [73,74]), DTT is steadily developing.

Another future direction spanning from DTT research is the development of bi-specific antibodies (BsAbs). The administration of two different biologics via infusion or subcutaneous injection can be uncomfortable for the patient, and it may cause severe localized or systemic reactions. To address this issue, BsAbs have been developed: antibodies containing two binding sites, directed toward two different but synergistic inflammatory molecules [75]. That is the case of molecules like V56B2 [76], which is able to specifically target both IL-23p19 and TNFα. Moreover, this molecule was engineered to be administered orally, adding to its convenience. Another recently developed BsAbs is FL-BsAb1/17, targeting IL-1β and IL-17A; it was compared to etanercept in a recent study [77], showing potential to become a new dual-target candidate for colitis treatment. Even though we do not know much of this approach yet, BsAb-focused studies have already yielded positive results, demonstrating potential for leaving a mark in the future of IBD management.

Additionally, an increasing number of IBD patients are turning to complementary medicine alongside conventional and emerging therapies, such as DTT. Adjunctive treatments, including herbal medicine [78] and, particularly, hyperbaric oxygen therapy [79,80], have shown potential benefits in certain cases and may serve as supportive strategies for complex pharmacological regimens like DTT. However, the current lack of robust evidence does not support their routine use in IBD management yet.

Still, many aspects of DTT remain unanswered. The understanding of DTT timing for utilization, as well as its role in induction versus maintenance phases, is of primary importance. As previously discussed, the most frequent indication for DTT in IBD is multi-drug refractory disease. However, implementing a contrary top–down approach is not always feasible due to the need of early diagnosis [81]. Additionally, the timing and methods of DTT discontinuation remain active topics of research. Theoretically, DTT could be used following a top–down approach, with an initial induction phase aimed at interrupting inflammation and preventing disease complications [82], followed by a maintenance phase using only the safer drug present in the chosen combination. However, the precise time of these phases is still unknown. Furthermore, it is worth considering whether the doses of individual medications in DTT could be optimized. Lower doses might maintain efficacy while potentially reducing the incidence of adverse effects, an idea that warrants dedicated investigation in future studies. Both these DTT timing issues correlate to another complex aspect: the costs. A top–down approach would be economically unsustainable for the healthcare budget of many countries. Although, with the development of biosimilar drugs—highly similar alternatives to reference biologics, offering comparable efficacy, safety, and immunogenicity [83]—this therapeutic strategy could improve its accessibility. Nevertheless, it is essential to state the necessity for large-scale, high-quality trials that could analyze the timing, phases, and cost effectiveness of DTT in order for it to become standard clinical practice.

Furthermore, there are two main issues in present DTT research: the heterogeneity of data and the scarcity of high-quality trials, like RCTs. The available studies regarding DTT take diverse approaches in analyzing their data. Some focus on specific drug combinations, categorizing results based on therapies combinations, while others organize their findings by disease type, examining whether the combinations were used in UC or CD. The majority of studies available do not focus on a single disease entity; instead, they often include mixed cohorts encompassing patients with various forms of IBD. While this approach provides broader insights into the application of combination therapies across the IBD spectrum, it limits the ability to draw disease-specific conclusions regarding efficacy and safety. This underscores the need for studies that evaluate the impact of such therapeutic strategies within distinct IBD subgroups to better tailor treatment approaches to the unique pathophysiology and clinical courses of UC and CD. Moreover, the majority of DTT data come from case series and small cohorts, limiting the quality of available findings.

The treat-to-target strategy and personalized, tailored therapy are the modern cornerstones of IBD management [84,85]. In order to ensure optimal disease control and long-lasting periods of remission, every treatment strategy should be globally standardized. Many questions remain unanswered in the field of DTT, restricting it from widely spread utilization. To refine the application of DTT in clinical practice, further large-scale, high-quality trials are needed. Nevertheless, continuous progress in these areas holds the potential to enhance patient outcomes and redefine IBD management.

Glimpsing into the future, there are several ongoing clinical trials regarding DTT (Table 3), including CD [86,87,88,89] and UC patients [90,91,92,93]. Across all these ongoing and planned studies, the indication for DTT initiation is constant: previous failure to multiple biologics or SMDs therapies.

## 6. Conclusions

In the evolving landscape of IBD management, DTT represents a compelling avenue to transcend the therapeutic ceiling. By simultaneously targeting complementary immune pathways, this strategy addresses the intricate and multifaceted nature of IBD. Emerging evidence, including promising real-world data and randomized clinical trials, underscores the potential of these innovative combinations in overcoming refractory disease and improving remission rates. However, the journey toward standardizing dual therapy is just beginning, and as research deepens, the focus should remain on personalized, precision medicine, harnessing synergy between agents while ensuring safety and cost effectiveness. The horizon holds promise for transforming and redefining success in IBD treatment.

## Figures and Tables

**Table 1:** Selected evidence of DTT efficacy in IBD.

Study	Study Design	Population	Combination Therapy	Efficacy Outcomes & Results
VEGA study (2023) [31]	Randomized, double-blind, controlled, proof-of-concept trial	214 moderate to severe UC patients, anti-TNF and anti-IL-12/23 naïve	Guselkumab + golimumab	At week 12: clinical response (83% in DTT vs. 61% golimumab and 75% guselkumab); clinical remission (37% in DTT).
Sands et al. (2007) [42]	Randomized controlled trial	79 CD patients with infliximab therapy failure	Infliximab + natalizumab	Mean CDAI reduction (37.7 in combination vs. 3.5 in placebo). Clinical remission: 46% DTT vs. 41% monotherapy.
EXPLORER trial (2024) [50]	Open-label trial	55 moderate to severe CD patients	Vedolizumab + adalimumab + MTX	Endoscopic remission (34.5% at 26 weeks).
Ahmed et al. (2022) [32]	Systematic review with meta-analysis	211 CD + 68 UC patients	TNF antagonist + anti-integrin; TNF antagonist + ustekinumab; TNF antagonist + tofacitinib; vedolizumab + ustekinumab; vedolizumab + tofacitinib; ustekinumab + tofacitinib	Over a median follow-up of 32 weeks: clinical remission 59% and endoscopic remission 34%.
Berinstein et al. (2023) [33]	Systematic review	288 UC and CD patients	TNF antagonist + anti-integrin; vedolizumab + ustekinumab; vedolizumab + tofacitinib; anti-TNF + tofacitinib; anti-TNF + ustekinumab; ustekinumab + tofacitinib	Clinical response (56%) and clinical remission (44% for UC).
Yang et al. (2020) [44]	Retrospective study	22 refractory CD patients	Vedolizumab + ustekinumab	Endoscopic remission (26%); clinical response (50%).
Miyatani et al. (2024) [49]	Case series	10 refractory CD patients	Ustekinumab +upadacitinib	Clinical remission (83%); improvement in EIM symptoms (75%).

**Table 2:** Selected evidence of DTT safety in IBD.

Study	Study Design	Population	Combination Therapy	Safety Data
VEGA trial (2023) [31]	Randomized, double-blind, controlled, proof-of-concept trial	214 moderate to severe UC patients, anti-TNF and anti-IL-12/23 naïve	Guselkumab + golimumab	At week 50: AEs 63% in DTT, 76% in golimumab, and 65% in guselkumab; infections 31% in DTT, 32% in golimumab, and 24% in guselkumab; serious infections, 3% in DTT, golimumab, and guselkumab groups.
Sands et al. (2007) [42]	Randomized controlled trial	79 CD patients with infliximab therapy failure	Infliximab + natalizumab	Comparable outcomes between monotherapy and DTT groups; only 1 case of SAEs in both groups; no opportunistic infections or malignancies.
EXPLORER trial (2024) [50]	Open-label trial	55 moderate to severe CD patients	Vedolizumab + adalimumab + methotrexate	AEs in 87.3%, and SAEs in only 10.9%, including one case of peritonitis and rectal abscess.
Ahmed et al. (2022) [32]	Systematic review and metanalysis	211 CD + 68 UC patients	TNF antagonist + anti-integrin; TNF antagonist + ustekinumab; TNF antagonist + tofacitinib; vedolizumab + ustekinumab; vedolizumab + tofacitinib; ustekinumab + tofacitinib	Over a median follow-up of 32 weeks: AEs in 31%; SAEs in 6.5%; infections 20%, and malignancies 2%.
Goessens et al. (2021) [57]	Retrospective study	40 UC and 58 CD patients	Anti-TNF + anti-integrin; anti-integrin + anti-TNF; anti-IL + anti-integrin; SMDs + anti-integrin	Serious AEs ~10%, severe infections often linked to associated corticosteroid use.
Miyatani et al. (2024) [49]	Case series	10 refractory CD patients	Ustekinumab + upadacitinib	AEs in 40%, including mild infections; 1 case of intestinal obstruction.
Gilmore et al. (2021) [56]	Case series	5 severe refractory UC patients	Infliximab + tofacitinib	1 case of varicella zoster; no thromboembolism or severe infections.

**Table 3:** Selected currently active clinical trials regarding DTT in IBD, as listed on clinicaltrials.gov (accessed on 7 January 2025).

Clinical Trials ID	Title/Objective	IBD Type	Therapy Combination	Study Design	Status	Target Population
NCT05242484 [91]	Guselkumab + golimumab in moderate to severe UC	UC	Guselkumab + golimumab	Phase 2b, randomized, placebo controlled	Ongoing	Patients with inadequate response to biologic therapies
NCT06095128 [90]	Vedolizumab + tofacitinib in moderate to severe UC	UC	Vedolizumab + tofacitinib	Phase 4, open label, multicenter	Ongoing	Patients with loss of response or intolerance to biologics
NCT05242471 [89]	Guselkumab + golimumab in moderate to severe CD	CD	Guselkumab + golimumab	Phase 2b, randomized, placebo controlled	Ongoing	Patients with active CD who failed advanced therapies
NCT06045754 [86]	Vedolizumab + adalimumab/ustekinumab in moderate to severe CD	CD	Vedolizumab + adalimumab/vedolizumab + ustekinumab	Phase 4, open label	Ongoing	Biologic-naïve or experienced patients with moderate to severe CD
NCT06520397 [87]	Upadacitinib + ustekinumab vs. intensified ustekinumab in CD	CD	Upadacitinib + ustekinumab	Randomized, controlled, multicenter	Planned	Patients with insufficient response to standard-dose Ustekinumab
NCT06227910 [88]	Vedolizumab + upadacitinib vs. vedolizumab monotherapy in moderate to severe CD	CD	Vedolizumab + upadacitinib	Phase 3b, randomized, placebo controlled	Ongoing	Patients with moderate to severe CD and prior biologic failure
NCT06453317 [93]	Ustekinumab + infliximab vs. either monotherapy in moderate to severe UC	UC	Ustekinumab + infliximab	Phase 2, open label	Planned	Adult, biologic-naïve patients with moderate to severe UC
NCT06095596 [92]	Upadacitinib + vedolizumab vs. either monotherapy in moderate to severe UC	UC	Upadacitinib + vedolizumab	Randomized, controlled, multicenter	Ongoing	Adult patients with moderate to severe UC

## Abbreviations

The following abbreviations are used in this manuscript:

IBDs	Inflammatory bowel diseases
UC	Ulcerative colitis
CD	Crohn’s disease
SMDs	Small-molecule drugs
JAK	Janus kinase
S1P	Sphingosine 1-phosphate
DTT	Dual-targeted therapy
EIMs	Extraintestinal manifestations
RCTs	Randomized controlled trials
TNF	Tumor necrosis factor
ECCO	European Crohn’s and Colitis Organization
CDAI	Crohn’s Disease Activity Index
IBDQ	Inflammatory Bowel Disease Questionnaire
SES-CD	Simple Endoscopic Score for Crohn’s disease
PRO-2	Crohn’s disease patient-reported outcome-2 score
CRP	C-reactive protein
CI	Confidence interval
AEs	Adverse events
SAEs	Severe adverse events
IMIDs	Immune-mediated inflammatory diseases
RA	Rheumatoid arthritis
SLE	Systemic lupus erythematosus
ACR 20/50	American College of Rheumatology 20 and 50 Response Criteria
BsAbs	Bi-specific antibodies
